# Clinical implications of ADHD, ASD, and their co-occurrence in early adulthood—the prospective ABIS-study

**DOI:** 10.1186/s12888-023-05298-3

**Published:** 2023-11-16

**Authors:** Andrea Lebeña, Åshild Faresjö, Tomas Faresjö, Johnny Ludvigsson

**Affiliations:** 1 Division of Pediatrics, Department of Biomedical and Clinical Sciences, Linköping University, Linköping, Sweden; 2 Department of Medicine and Health, Community Medicine, Linköping University, Linköping, Sweden; 3 Crown Princess Victoria Children’s Hospital, Region Östergötland, Linköping, Sweden

**Keywords:** ADHD, ASD, Co-occurrence, ABIS, Impact on health, Psychosocial vulnerabilities, Young adults

## Abstract

**Background:**

Attention-deficit/hyperactivity disorder (ADHD) and autism spectrum disorder (ASD) are childhood-onset disorders associated with functional and psychosocial impairments that may persist into adulthood, leading to serious personal and societal costs.

**Objective:**

This study aimed to examine the socio-economic difficulties, physical and mental comorbidities, and psycho-social vulnerabilities associated with ADHD, ASD, and their co-occurrence among young adults.

**Methods:**

16 365 families with children born 1997–1999, were involved in the prospective population-based ABIS study (All Babies in Southeast Sweden). A total of 6 233 ABIS young adults answered the questionnaire at the 17–19-year follow-up and were included in this case–control study. Diagnoses of ADHD and ASD from birth up to 17 years of age were obtained from the Swedish National Diagnosis Register. *N*=182 individuals received a single diagnosis of ADHD, *n*=78 of ASD, and *n*=51 received both diagnoses and were considered the co-occurrence group. Multiple multinomial logistic regression analyses were performed.

**Results:**

In the univariate analyses all three conditions were significantly associated with concentration difficulties, worse health quality, lower socio-economic status, lower faith in the future, less control over life, and lower social support. In the adjusted analyses, individuals with ADHD were almost three-times more likely to have less money compared with their friends (aOR 2.86; *p* < .001), experienced worse sleep quality (aOR 1.50; *p* = .043) and concentration difficulties (aOR 1.96; *p* < .001). ASD group were two-fold more likely to experience concentration difficulties (aOR 2.35; *p* = .002) and tended not to have faith in the future (aOR .63; *p* = .055), however, showed lesser risk-taking bahaviours (aOR .40; *p* < .001). Finally, the co-occurrence was significantly associated with unemployment (aOR 2.64; *p* = .007) and tended to have a higher risk of autoimmune disorders (aOR 2.41; *p* = .051), however, showed a 51% lower risk of stomach pain (aOR .49; *p* = .030).

**Conclusions:**

All these conditions significantly deteriorated several areas of life. ADHD/ASD co-occurrence is a heavy burden for health associated with several psychosocial vulnerabilities, that shared a similar morbidity pattern with ADHD although showed less risk cognitive and behavioral profile, similar to the ASD group. Long-term follow-up and support for individuals with these conditions over the life course are crucial.

**Supplementary Information:**

The online version contains supplementary material available at 10.1186/s12888-023-05298-3.

## Introduction

Attention-deficit/hyperactivity disorder (ADHD) and autism spectrum disorder (ASD) are common childhood-onset neurodevelopmental disorders [1, 2] generally persisting into adulthood [3–5]. The challenges for these persons are twofold: they face age-related effects experienced by the general population, such as new social challenges and biological and emotional transition to adulthood, alongside disorder-specific effects [6].

The phenotypes of neurodevelopmental disorders (NDDs) are heterogeneous, and their complexity is compounded by high comorbidity rates with several conditions (i.e., gastrointestinal disturbances, congenital anomalies, and immunological disorders) [7]. In previous studies, ADHD and ASD have been associated with coexisting psychiatric and neurological conditions, such as oppositional and conduct disorders, tic disorders, epilepsy, depression, anxiety, and substance use disorders [8, 9]. Moreover, both disorders have been found to be associated with psychosocial functional impairments and a range of adverse outcomes in patients and their families [4, 5, 10, 11]. Children and adults with ADHD or ASD often experience emotional and social difficulties, which also negatively impact their quality of life [3–5].

It has also been shown that psychological, physical, and sexual forms of abuse and household dysfunction such as substance abuse, mental illness, and violence were associated with risk behaviours like binge drinking and smoking, poor health in general, and a higher risk of obesity, myocardial infarction, and stroke [12]. Risk-taking behaviours and unhealthy habits are mostly established during adolescence and are often carried into adulthood, having long-term effects on lifestyle and health [13]. Finally, several studies have suggested that ASD and ADHD may be even associated with an increased risk of mortality due to both natural and non-natural causes [14–16].

Despite the growing body of research pointing at the impact of ADHD and ASD on health and quality of life, little is known regarding their co-occurrence, which could be associated with greater impairment than a single condition and could be less responsive to standard treatments for either disorder. The current study aimed to examine the socio-economic difficulties, physical and mental comorbidities, and psycho-social vulnerabilities associated with ADHD, ASD, and especially their co-occurrence in early adulthood.

## Methods

### Study population

**Fig. 1:**
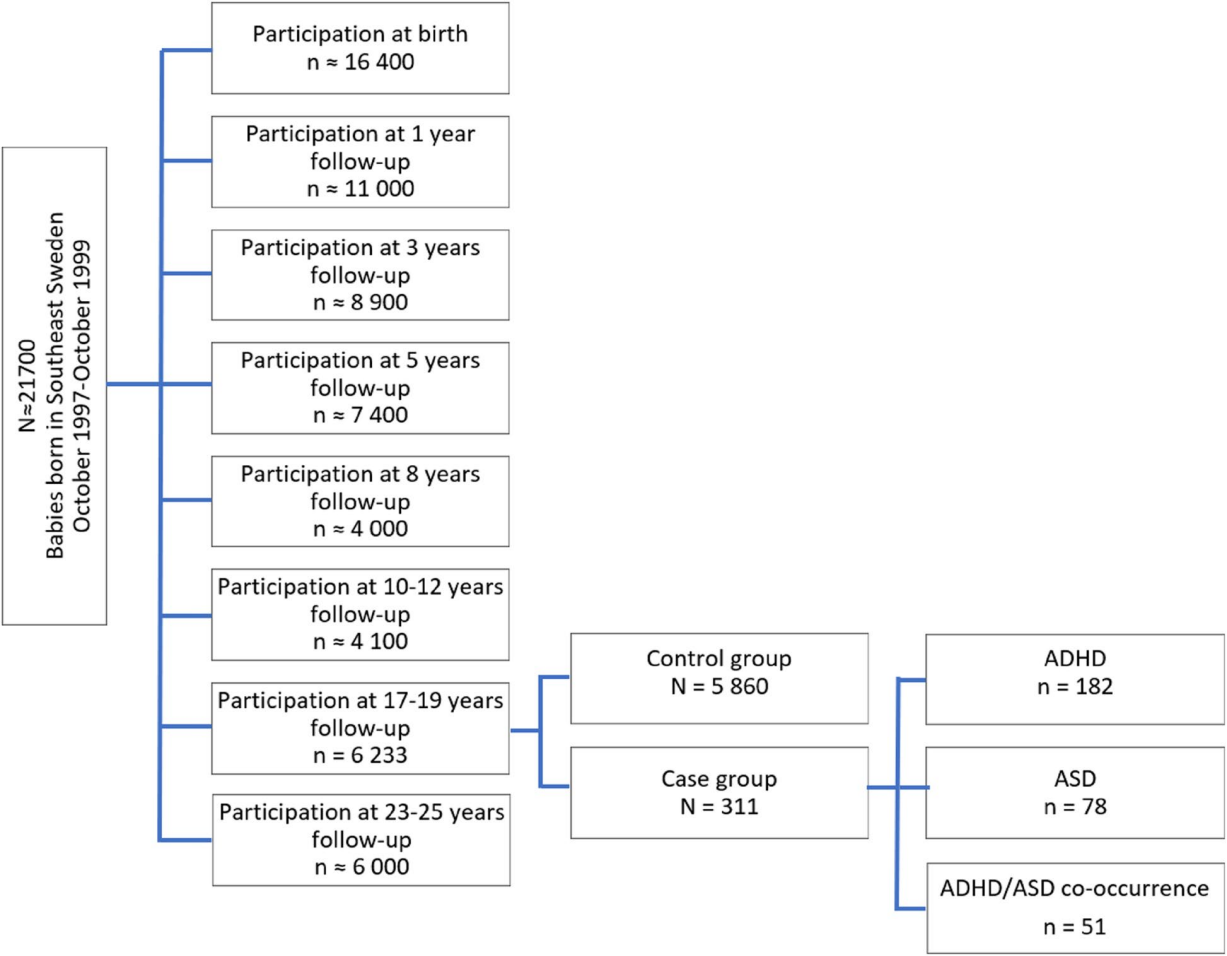
Study population flow-chart Definition of case and control groups based on the cumulative incidence rates for ADHD, ASD, and their co-occurrence from birth until 17 years of age. ADHD indicates attention-deficit/hyperactivity disorder, while ASD indicates autism spectrum disorder

This study includes data from the ABIS-Study (All Babies in Southeast Sweden), a longitudinal, population-based cohort study based on data collected from 16 365 families with children born between October 1997 and October 1999 in Southeast Sweden. ABIS-Study aims to investigate how environmental and genetic factors influence the development of immune-mediated diseases, which include ADHD and ASD, where immune mechanisms may play a role [17]. The children included in the ABIS-Study have been followed from birth onwards, and questionnaires data, biological samples, and register data of diseases (based on medical records) have been collected at birth and age of 1, 3, 5, 8, 10–12, 17–19, and 23–25 years. A total of 6 233 young adults who were included in the ABIS-Study at birth and answered the questionnaire at 17–19 years follow-up, were included in this prospective case–control sub study (Fig. 1).

### Diagnosis of ADHD, ASD, and their co-occurrence

The diagnoses of ADHD, ASD, and their co-occurrence were obtained from birth until 17 years of age for the 17–19 years follow-up participants (*n* = 6 233), by cross-linking with the Swedish National Patient Register (NPR), containing all hospital inpatients (since 1973) and outpatients (since 2001) International Classification of Diseases (ICD-8 to ICD-10) based on doctor-set diagnoses [18]. According to ICD-10, F90 (F90.0, F90.1, F90.8, and F90.9) and F84 (F84.0, F84.1, F84.2, F84.3, F84.4, F84.5, F84.8, and F84.9) were the diagnostic codes used for ADHD and ASD, respectively. Those participants who got a unique diagnosis of ADHD (*n* = 182), those who received a unique ASD diagnosis (*n* = 78), and those who got both diagnoses (ADHD and ASD co-occurrence) (*n* = 51), according to the NPR, are the three-case groups. The rest of the study population constitutes the control group (*N* = 5 860) (Fig. 1).

The parents were given oral and written information before giving informed consent to participate in the study. The ABIS study was approved by the research ethics committees at Linköping University (Dnr 96–287, Dnr 99–321, and Dnr 03–092) and Lund University (LU 83–97) in Sweden, and connection of the ABIS registers to National registers was approved by the Research Ethics Committee in Linköping (Dnr 2013/253–32). All methods were carried out following relevant guidelines and regulations.

### Measures

The items from the web survey answered at 17–19 years of age (see Additional file 1), were categorized into four major areas:

#### Socio-economic indicators

Included if they have enough money to do the same thing as their friends, and questions regarding occupation (studying, working or unemployed).

#### Health-related factors

Comprised questions regarding health quality (ranged from 1 very poor to 5 excellent), exercise until get sweaty (dichotomic), weekdays and weekends screen time exposure (categorized in less or more than 4hs per day, according to The American Academy of Pediatrics—AAP) [19], sleep quality (ranged from 1 very poor to 5 excellent), headache, stomach, and joint pain (ranged from 1 never to 5 almost every day), if they have been or being severely ill (dichotomic), if they have allergies, and BMI (underweight < 18.36, normal 18.37–26.35, overweight 26.36–30.11, or obese > 30.12). Doctor-set diagnoses of autoimmune diseases: celiac disease, psoriasis, immune thrombocytopenic purpura, hypothyroidism, thyrotoxicosis (hyperthyroidism), autoimmune thyroiditis, type 1 diabetes mellitus, arteritis, Crohn’s disease, ulcerative colitis, vitiligo, juvenile arthritis, Kawasaki syndrome, and Sjögren syndrome, were obtained by cross-linking with the Swedish National Patient Register (NPR).

#### Psychosocial vulnerability

Involved questions about faith in the future, control over life, perceived stress during the last month (ranged from 1 not at all to 10 very much), and being bullied (ranged from 1 never to 5 always). If they feel down or depressed, worried or anxious, and concentration difficulties (ranged from 1 never to 5 almost every day) were also added. Questions regarding job or academic feelings/performance*,* and social support (from friends, family, or school) were included as well. It also involved if the participants had been exposed to serious life events in the last two years, including death or severe illness in the family (death of parent, sibling, or grandparents, and severe illness within the family), unstable family situation (many conflicts between adults, divorced or separated parents, sole custody with regular or no/sporadic contact with the non-custodial parent, new adults in the family, new children in the family, contact with a supportive family), contact with social authorities for support, if they were sexually or physically abused (by an adult or peer), and robbery victim. An index of stressful life events was developed based on the cumulative frequency of the described stressful events (none, one or two, more than three).

#### Risk-taking behaviours and perceptions of risks

The questionnaire included 5 dichotomic items regarding tobacco smoking, e-cigarette use, hashish/marijuana smoking, snuff use, and alcohol consumption. An index with a max. score of 5 points was made and a higher score corresponded to many unhealthier/risk-taking behaviours. The questionnaire also included other 5 items that assessed particpants´perceptions of the above-mentioned risk-taking behaviours (eg. “imagine someone that smokes 2–3 times/day: how harmful do you think this is for health?”). All items ranged from 1 (not harmful at all) to 5 (extremely harmful), the index had a max. score of 25. Scores from 5 to 15 were considered slightly/moderately harmful, while scores between 16 and 25 were considered extremely/quite harmful.

### Statistical analyses

All statistical analyses were performed in SPSS software version 28.0 (IBM SPSS Inc., Chicago, IL, USA). Dichotomous variables were presented as frequencies and percentages, and differences between groups were assessed using the Chi-squared test. A *p*-value ≤ 0.05 was considered statistically significant, and multiple comparisons between the three case-groups were adjusted using Bonferroni correction (Tables 1, 2, 3 and 4). A comparison was made between those who participated in the 17–19 year follow-up and those who did not to evaluate the risk of skewness in participation over time. Identification of statistically independent discriminators used a backward elimination algorithm in which all univariately statistically significant discriminators (Unadjusted model – Table 5) were entered into a single full model in the multiple multinomial logistic regression analyses (Adjusted model – Table 5). Effect sizes were reported as odds ratios (OR) within 95% confidence intervals (95% CI) and 2-tailed *p*-values.

## Results

### Socio-economic indicators

All case-groups showed lower participation in the 17–19 years follow-up compared to the controls. Females constitute 36.5% of NDDs cases, 48.4% of ADHD, 37.2% of ASD, and 37.3% of the co-occurrence group, were women. All three conditions reported having less money than friends, while ASD and the co-occurrence groups were more likely to be unemployed (Table 1).

**Table 1:** Socio-economic characteristics of case-groups and controls

	**ADHD**^**a**^** (*n* = 182)**	***P* value**	**ASD**^**b**^** (*n* = 78)**	***P* value**	**Co-occurrence**^**c**^** (*n* = 51)**	***P* value**	**Controls (*n* = 5860)**	**Case groups comparisons**
**Participation 17–19 year follow-up**		** < .001**		**.025**		**.003**		a-b*
Yes	182 (25.2%)		78 (31.6%)		51 (27.9%)		5860 (38.6%)	
No	541 (74.8%)		169 (68.4%)		132 (72.1%)		9340 (61.4%)	
**Sex**		.093		**.002**		**.013**		
Male	94 (51.6%)		49 (62.8%)		32 (62.7%)		2657 (45.3%)	
Female	88 (48.4%)		29 (37.2%)		19 (37.3%)		3202 (54.7%)	
**Occupation**		.219		**.001**		** < .001**		a-c**
Study/working	150 (88.2%)		61 (80.3%)		36 (72.0%)		5147 (91.0%)	
Unemployed	20 (11.8%)		15 (19.7%)		14 (28%)		510 (9.0%)	
**Enough money compared with friends**		** < .001**		**.001**		** < .001**		
Always/often	120 (76.9%)		62 (84.9%)		36 (78.3%)		5120 (94.0%)	
Never/seldom	36 (23.1%)		11 (15.1%)		10 (21.7%)		325 (6.0%)	

*Abbreviations: ADHD* Attention-deficit/hyperactivity disorder, *ASD* Autism spectrum disorder. Values are presented as absolute numbers (percentage of cases/controls). All *p*-values were calculated for each case group against the control group from Chi-squared test. * *P* < .05, ** *P* < .01 and *** *P* < .001. Case-groups comparisons column indicates between which case-groups were found statistically significant differences (a: ADHD, b: ASD, c: Co-occurrence), and its magnitude

### Health-related outcomes

All three case-groups reported worse health quality compared with the control group. The ADHD group reported having somatic complaints (stomach and joint pain) more regularly, lower physical activity, and worse sleep quality than the control group did. ASD group reported having severe illness in the last two years, lower physical activity, and were more likely to be overweight/obese. The ASD group also reported longer screen exposure (> 4 h/day) than the control group. The co-occurrence group was more likely to have a severe illness and autoimmune diseases, tended to be underweight or overweight/obese, and reported worse sleep quality than the control group did. The co-occurrence group also reported longer screen exposure (> 4 h/day) during weekends but was not significant (Table 2).

**Table 2:** Health outcomes among case-groups and controls

	**ADHD**^**a**^** (*n* = 182)**	***P* value**	**ASD**^**b**^** (*n* = 78)**	***P* value**	**Co-occurrence**^**c**^** (*n* = 51)**	***P* value**	**Controls (*n* = 5860)**	**Case groups comparisons**
**Health quality**		** < .001**		**.005**		**.003**		
Excellent/very good/good	109 (78.4%)		53 (76.8%)		29 (72.5%)		4405 (87.9%)	
Quite bad/bad	30 (21.6%)		16 (23.2%)		11 (27.5%)		608 (12.1%)	
**Severe illness**		.739		**.008**		**.004**		a-c*
Yes	10 (5.5%)		9 (11.5%)		7 (13.7%)		290 (4.9%)	
No	172 (94.5%)		69 (88.5%)		44 (86.3%)		5570 (95.1%)	
**Autoimmune disease**		.857		.735		**.017**		
Yes	9 (4.9%)		3 (3.8%)		6 (11.8%)		273 (4.7%)	
No	721 (95.5%)		255 (93.8%)		173 (92.0%)		5587 (95.3%)	
**BMI**		.501		**.002**		** < .001**		a-c*
Underweight	10 (6.8%)		5 (8.1%)		7 (15.9%)		251 (4.9%)	
Normal	114 (77.6%)		39 (62.9%)		26 (59.1%)		4146 (80.7%)	
Overweight/obese	23 (15.6%)		18 (29.0%)		11 (25.0%)		742 (14.4%)	
**Allergies**		.802		.172		.407		
Yes	61 (44.9%)		26 (37.7%)		21 (52.5%)		2290 (45.9%)	
No	75 (55.1%)		43 (62.3%)		19 (47.5%)		2695 (54.1%)	
**Headache**		.360		.727		.589		
Never/seldom	51 (35.4%)		26 (37.1%)		19 (43.2%)		2029 (39.2%)	
Sometimes/regularly	93 (64.6%)		44 (62.9%)		25 (56.8%)		3148 (60.8%)	
**Stomach pain**		**.005**		.111		.178		a-b** a-c**
Never/seldom	46 (31.7%)		37 (52.9%)		24 (53.3%)		2238 (43.3%)	
Sometimes/regularly	99 (68.3%)		33 (47.1%)		21 (46.7%)		2926 (56.7%)	
**Joint pain**		**.001**		.171		.172		
Never/seldom	98 (67.6%)		49 (72.1%)		31 (70.5%)		4067 (78.9%)	
Sometimes/regularly	47 (32.4%)		19 (27.9%)		13 (29.5%)		1088 (21.1%)	
**Exercise until get sweaty**		**.008**		**.028**		.152		
Yes	111 (71.6%)		51 (69.9%)		33 (71.7%)		4351 (80.2%)	
No	44 (28.4%)		22 (30.1%)		13 (28.3%)		1074 (19.8%)	
**Sleep quality**		** < .001**		.863		** < .001**		a-b* b-c*
Excellent/very good/good	100 (66.2%)		58 (82.9%)		30 (65.2%)		4382 (83.6%)	
Quite bad/bad	51 (33.8%)		12 (17.1%)		16 (34.8%)		858 (16.4%)	
**Screen exposure (weekdays)**		.286		.122		.782		
0-4h	51 (33.3%)		20 (28.6%)		16 (35.6%)		2021 (37.6%)	
> 4h	102 (66.7%)		50 (71.4%)		29 (64.4%)		3359 (62.4%)	
**Screen exposure (weekends)**		.190		**.001**		.087		a-b*
0-4h	61 (39.9%)		19 (26.0%)		15 (32.6%)		2430 (45.2%)	
> 4h	92 (60.1%)		54 (74.0%)		31 (67.4%)		2944 (54.8%)	

*Abbreviations*: *ADHD* Attention-deficit/hyperactivity disorder, *ASD* Autism spectrum disorder. Values are presented as absolute numbers (percentage of cases/controls). All *p*-values were calculated for each case group against the control group from Chi-squared test. * *P* < .05, ** *P* < .01 and *** *P* < .001. Case-groups comparisons column indicates between which case-groups were found statistically significant differences (a: ADHD, b: ASD, c: Co-occurrence), and its magnitude

### Psychosocial vulnerability

All three case-groups reported having contacted social authorities for support, regular concentration difficulties, lower faith in the future, and a lack of social support, compared with the control group. The ADHD and the co-occurrence groups were more likely to experienced 3 or more serious life events in the last two years, however, those events were more violence-related (physical abuse by an adult or peer and robberies) in the ADHD group, while death/illness in the family and unstable family situation where predominantly associated with the co-occurrence group. Both groups also indicated worse job or academic feelings/performance, frequent anxious and depressed feelings, and being bullied (did not rich the significance in the ADHD group). The ADHD group also reported greater perceived stress, and less control over life than the control group did. The ASD group, like the ADHD group, reported less control over life (Table 3).

**Table 3:** Psychosocial vulnerabilities among case-groups and healthy controls

	**ADHD**^**a**^** (*n* = 182)**	***P* value**	**ASD**^**b**^** (*n* = 78)**	***P* value**	**Co-occurrence**^**c**^** (*n* = 51)**	***P* value**	**Controls (*n* = 5860)**	**Case groups comparisons**
**Serious life event index**		** < .001**		.262		** < .001**		
None	100 (54.9%)		42 (53.8%)		22 (43.1%)		3364 (57.4%)	
1–2	65 (35.7%)		31 (39.7%)		20 (39.2%)		2310 (39.4%)	
> 3	17 (9.3%)		5 (6.4%)		9 (17.6%)		186 (3.2%)	
**Death/illness family**		.691		.934		**.015**		
Yes	53 (29.1%)		22 (28.2%)		22 (43.1%)		1628 (27.8%)	
No	129 (70.9%)		56 (71.8%)		29 (56.9%)		4232 (72.2%)	
**Unstable family situation**		.113		.240		**.013**		
Yes	41 (22.5%)		18 (23.1%)		16 (31.4%)		1051 (17.9%)	
No	141 (77.5%)		60 (76.9%)		35 (68.6%)		4809 (82.1%)	
**Sexually abused**		.079		.766		.756		
Yes	10 (5.5%)		2 (2.6%)		2 (3.9%)		185 (3.2%)	
No	172 (94.5%)		76 (97.4%)		49 (96.1%)		5675 (96.8%)	
**Physically abuse**		** < .001**		.794		.380		
Yes	14 (7.7%)		2 (2.6%)		2 (3.9%)		125 (2.1%)	
No	168 (92.3%)		76 (97.4%)		49 (96.1%)		5735 (97.9%)	
**Robbery victim**		** < .001**		.740		.972		
Yes	12 (6.6%)		2 (2.6%)		1 (2.0%)		119 (2.0%)	
No	170 (93.4%)		76 (97.4%)		50 (98.0%)		5741 (98.0%)	
**Social authorities support**		**.003**		**.003**		** < .001**		a-c*
Yes	7 (3.8%)		4 (5.1%)		6 (11.8%)		75 (1.3%)	
No	175 (96.2%)		74 (94.9%)		45 (88.2%)		5785 (98.7%)	
**Performance at school/work**		** < .001**		.440		**.023**		
Bad	13 (9.2%)		3 (5.6%)		4 (11.8%)		181 (3.7%)	
Good	55 (38.7%)		13 (24.1%)		13 (38.2%)		1534 (31.4%)	
Very good	74 (52.1%)		38 (70.4%)		17 (50.0%)		3176 (64.9%)	
**Faith in the future**		**.004**		** < .001**		** < .001**		
Hopeless	13 (8.1%)		10 (14.1%)		5 (10.9%)		204 (3.7%)	
Moderate	52 (32.3%)		27 (38.0%)		21 (45.7%)		1515 (27.6%)	
Hopeful	96 (59.6%)		34 (47.9%)		20 (43.5%)		3765 (68.7%)	
**Perceived stress**		**.002**		.248		.836		a-b*
Low	34 (24.6%)		21 (31.3%)		9 (20.5%)		1195 (24.3%)	
Moderate	47 (34.1%)		32 (47.8%)		22 (50.0%)		2312 (47.0%)	
High	57 (41.3%)		14 (20.9%)		13 (29.5%)		1416 (28.8%)	
**Control over life**		**.007**		** < .001**		.071		
No control	21 (15.0%)		15 (22.1%)		7 (16.7%)		380 (7.7%)	
Moderate	57 (40.7%)		30 (44.1%)		19 (45.2%)		2136 (43.4%)	
Full control	62 (44.3%)		23 (33.8%)		16 (38.1%)		2404 (48.9%)	
**Bully victim**		.060		.660		** < .001**		b-c*
Never/seldom	131 (90.3%)		65 (92.9%)		36 (80.0%)		4885 (94.1%)	
Sometimes/often/always	14 (9.7%)		5 (7.1%)		9 (20.0%)		306 (5.9%)	
**Social support**		**.019**		**.042**		**.005**		
Yes	130 (89.7%)		62 (88.6%)		38 (84.4%)		4890 (94.3%)	
No	15 (10.3%)		8 (11.4%)		7 (15.6%)		296 (5.7%)	
**Feeling depressed**		**.001**		.142		**.029**		
Never/seldom/sometimes	91 (65.5%)		48 (69.6%)		25 (62.5%)		3868 (77.1%)	
Regularly	48 (34.5%)		21 (30.4%)		15 (37.5%)		1151 (22.9%)	
**Feeling worried/anxious**		**.003**		.255		**.011**		
Never/seldom/sometimes	79 (56.8%)		43 (62.3%)		20 (50.0%)		3441 (68.7%)	
Regularly	60 (43.2%)		26 (37.7%)		20 (50.0%)		1566 (31.3%)	
**Concentration difficulties**		** < .001**		** < .001**		**.022**		
Never/seldom/sometimes	59 (42.4%)		35 (50.7%)		22 (55.0%)		3583 (71.5%)	
Regularly	80 (57.6%)		34 (49.3%)		18 (45.0%)		1429 (28.5%)	

*Abbreviations*: *ADHD* Attention-deficit/hyperactivity disorder, *ASD* Autism spectrum disorder. Values are presented as absolute numbers (percentage of cases/controls). All *p*-values were calculated for each case group against the control group from Chi-squared test. * *P* < .05, ** *P* < .01 and *** *P* < .001. Case-groups comparisons column indicates between which case-groups were found statistically significant differences (a: ADHD, b: ASD, c: Co-occurrence), and its magnitude

### Risk-taking behaviours and perceptions of risks

Tobacco, e-cigarettes, hashish/marijuana smoking, and snuff use, were more frequently reported among the ADHD group. ASD group reported instead e-cigarettes and hashish/marijuana smoking, but also alcohol consumption to a lesser extent than the control group did. The co-occurrence group was also less likely to alcohol consumption compared with the control group. Regarding perceptions of risks, tobacco and e-cigarettes smoking every day, and hashish/marijuana smoking and snuff use every week, were considered less harmful among the ADHD group compared to the control group (Table 4).

**Table 4:** Risk-taking behaviours and perceptions among case-groups and healthy controls

	**ADHD**^**a**^** (*n* = 182)**	***P* value**	**ASD**^**b**^** (*n* = 78)**	***P v*alue**	**Co-occurrence**^**c**^** (*n* = 51)**	***P* value**	**Controls (*n* = 5860)**	**Case-groups comparisons**
**Smoking**		** < .001**		.150		.129		a-b*** b-c*
Yes	36 (24.3%)		2 (2.9%)		6 (13.3%)		385 (7.4%)	
No	112 (75.7%)		68 (97.1%)		39 (86.7%)		4839 (92.6%)	
**e-cigarette smoking**		** < .001**		**.012**		.765		a-b*** a-c*
Yes	67 (48.6%)		10 (15.6%)		10 (27.8%)		1438 (30.1%)	
No	71 (51.4%)		54 (84.4%)		26 (72.2%)		3344 (69.9%)	
**Hashish/marijuana smoking**		**.012**		**.038**		.470		a-b** b-c*
Yes	32 (22.2%)		4 (5.8%)		8 (18.6%)		757 (14.7%)	
No	112 (77.8%)		65 (94.2%)		35 (81.4%)		4399 (85.3%)	
**Snuff use**		** < .001**		.392		.500		a-b*
Yes	29 (19.6%)		5 (7.1%)		6 (13.3%)		536 (10.3%)	
No	119 (80.4%)		65 (92.9%)		39 (86.7%)		4687 (89.7%)	
**Alcohol consumption**		.797		** < .001**		** < .001**		a-b** a-c***
Yes	126 (86.9%)		48 (69.6%)		27 (62.8%)		4526 (87.6%)	
No	19 (13.1%)		21 (30.4%)		16 (37.2%)		640 (12.4%)	
**Smoking everyday**		** < .001**		.210		.631		
Extremely/quite harmful	117 (79.6%)		58 (84.1%)		41 (91.1%)		4638 (88.9%)	
Not/slightly/moderately harmful	30 (20.4%)		11 (15.9%)		4 (8.9%)		582 (11.1%)	
**Smoking (e-cigarette) every day**		** < .001**		.411		.862		
Extremely/quite harmful	42 (30.4%)		26 (40.6%)		17 (47.2%)		2190 (45.8%)	
Not/slightly/moderately harmful	96 (69.6%)		38 (59.4%)		19 (52.8%)		2594 (54.2%)	
**Smoking (hashish/marijuana) every week**		**.034**		.931		.205		a-c*
Extremely/quite harmful	86 (59.3%)		45 (68.2%)		33 (76.7%)		3491 (67.7%)	
Not/slightly/moderately harmful	59 (40.7%)		21 (31.8%)		10 (23.3%)		1667 (32.3%)	
**Snuff use every week**		**.002**		.277		.875		
Extremely/quite harmful	61 (41.5%)		33 (47.8%)		25 (55.6%)		2841 (54.4%)	
Not/slightly/moderately harmful	86 (58.5%)		36 (52.2%)		20 (44.4%)		2383 (45.6%)	
**Alcohol consumption every week**		.693		.227		.063		b-c*
Extremely/quite harmful	55 (37.9%)		22 (32.4%)		23 (53.5%)		2042 (39.6%)	
Not/slightly/moderately harmful	90 (62.1%)		46 (67.6%)		20 (46.5%)		3120 (60.4%)	

Abbreviations: ADHD Attention-deficit/hyperactivity disorder, ASD Autism spectrum disorder. Values are presented as absolute numbers (percentage of cases/controls). All p-values were calculated for each case group against the control group from Chi-squared test. * P < .05, ** P < .01 and *** P < *.001. Case-groups comparisons column indicates between which case-groups were found statistically significant differences (a: ADHD, b: ASD, c: Co-occurrence), and its magnitude*

### Case-group comparisons

The ADHD group showed lower participation in the 17–19 year follow-up than ASD and reported lower unemployment rates than the co-occurrence group. The co-occurrence group, compared to the ADHD were more likely to be either underweight or overweight/obese and to report severe illness, while ADHD reported having stomach pain more frequently than ASD and the co-occurrence group. The ASD group reported longer screen exposure (during weekends) than the ADHD group, and better sleep quality than the ADHD and the co-occurrence group. In terms of vulnerability, the co-occurrence group reported having contact with social authorities to a greater extent than the ADHD group and being bullied more often compared to the ASD group. The ADHD group perceived higher stress levels than the ASD group did. What concerns about risk-taking behaviours, the ADHD group was more likely to smoke e-cigarettes and consume alcohol than ASD and the co-occurrence group, but also used snuff to a  greater extent than the ASD group. Oppositely, the ASD group reported tobacco and hashish/marijuana smoking to a lesser extent than the ADHD and the co-occurrence group. Regarding perceptions of risk-taking behaviours, the co-occurrence group considered smoking hashish/marijuana every week as more harmful than the ADHD group, and alcohol consumption every week than the ASD group (case-groups comparisons column, Tables 1, 2, 3 and 4).

### Statistically independent effects

Less money than friends, concentration difficulties, and bad sleep quality remain significant for ADHD in the multiple multinomial logistic regression analyses, while perceptions of risks showed a tendential effect (Table 5). Regarding the ASD group, concentration difficulties, and fewer risk-taking behaviours were the ones that remained significant in the multiple multinomial logistic regression analyses, together with a lower faith in the future, lower perceived stress levels, and less money than friends, all of them showed a tendential effect. Finally, being unemployed, and having less stomach pain were the ones statistically associated with the co-occurrence group, while having an autoimmune disease was tendential.

**Table 5:** Multinomial logistic regression analysis, effect sizes represented as odds ratios. Case groups compared to the control group

Unadjusted model^a^							Adjusted model^b^					
	**ADHD (*n* = 182) OR (95% CI)**	***P* value**	**ASD (*n* = 78) OR (95% CI)**	***P* value**	**Co-occurrence (*n* = 51) OR (95% CI)**	***P* value**	**ADHD (*n* = 182) OR (95% CI)**	***P* value**	**ASD (*n* = 78) OR (95% CI)**	***P* value**	**Co-occurrence (*n* = 51) OR (95% CI)**	***P* value**
**Socio-economic indicators**												
**Unemployed**	1.36 (.84–2.20)	.208	2.45 (1.38–4.35)	**.002**	3.83 (2.06–7.13)	** < .001**	1.02 (.60–1.74)	.928	1.32 (.69–2.52)	.407	2.64 (1.31–5.32)	**.007**
**Less money than friends**	4.11 (2.80–6.02)	** < .001**	2.75 (1.45–5.21)	**.002**	4.14 (2.01–8.53)	** < .001**	2.86 (1.82–4.51)	** < .001**	1.85 (.90–3.84)	.096	1.88 (.80–4.41)	.147
**Health-related outcomes**												
**Bad health quality**	1.89 (1.28–2.79)	**.002**	2.13 (1.23–3.69)	**.007**	2.69 (1.45–5.01)	**.002**	1.02 (.63–1.67)	.927	1.18 (.58–2.39)	.644	1.21 (.53–2.78)	.649
**Autoimmune disease**	1.06 (.54–2.10)	.857	.82 (.26–2.61)	.735	2.73 (1.15–6.45)	**.022**	.88 (.44–1.78)	.724	.79 (.24–2.55)	.689	2.41 (.99–5.83)	.051
**BMI**	1.05 (.74–1.49)	.783	1.72 (1.02–2.90)	**.040**	1.12 (.56–2.22)	.749	.93 (.66–1.31)	.688	1.43 (.89–2.32)	.142	.98 (.52–1.84)	.951
**Allergies**	.98 (.69–1.40)	.920	.75 (.46–1.21)	.236	1.28 (.68–2.39)	.437						
**Headache**	1.17 (.80–1.70)	.410	1.08 (.65–1.80)	.762	.88 (.49–1.58)	.680						
**Stomach pain**	1.49 (1.05–2.11)	**.027**	.70 (.41–1.18)	.178	.70 (.40–1.23)	.220	1.20 (.83–1.76)	.331	.65 (.35–1.22)	.175	.49 (.25-.93)	**.030**
**Joint pain**	1.64 (1.17–2.30)	**.004**	1.37 (.80–2.33)	.250	1.46 (.77–2.76)	.245	1.14 (.78–1.66)	.503	1.35 (.74–2.49)	.326	1.12 (.54–2.34)	.755
**No exercise**	1.48 (1.03–2.13)	**.036**	1.73 (1.04–2.88)	**.035**	1.45 (.75–2.79)	.267	1.06 (.71–1.58)	.772	1.19 (.67–2.14)	.548	.92 (.43–1.96)	.820
**Bad sleep quality**	2.36 (1.71–3.26)	** < .001**	1.13 (.60–2.14)	.704	2.59 (1.39–4.82)	**.003**	1.50 (1.01–2.22)	**.043**	.67 (.32–1.40)	.283	1.58 (.70–3.53)	.265
**Screen exposure weekdays (> 4h)**	1.18 (.86–1.63)	.296	1.52 (.91–2.54)	.112	1.25 (.68–2.31)	.474						
**Screen exposure weekends (> 4h)**	1.26 (.92–1.72)	.148	2.21 (1.29–3.79)	**.004**	1.80 (.96–3.38)	.065	1.08 (.78–1.50)	.629	1.59 (.90–2.84)	.111	1.43 (.76–2.72)	.269
**Psychosocial vulnerability**												
**Performance at work/school**	.66 (.49-.88)	**.006**	1.09 (.68–1.74)	.721	.60 (.35–1.01)	.056	.79 (.58–1.08)	.133	1.36 (.81–2.28)	.242	.71 (.40–1.24)	.223
**Faith in the future**	.71 (.55-.91)	**.007**	.48 (.34-.67)	** < .001**	.46 (.30-.69)	** < .001**	1.08 (.76–1.54)	.650	.63 (.40–1.01)	.055	.72 (.41–1.26)	.257
**Perceived stress**	1.21 (.98–1.49)	.069	.83 (.59–1.15)	.260	1.08 (.72–1.62)	.698	.92 (.71–1.19)	.527	.70 (.46–1.07)	.099	.79 (.49–1.28)	.334
**Control over life**	.78 (.61-.99)	**.048**	.55 (.38-.79)	**.001**	.63 (.42-.96)	**.031**	1.13 (.81–1.59)	.461	.76 (.46–1.25)	.282	1.19 (.65–2.20)	.571
**Being bullied**	1.55 (.83–2.87)	.162	1.13 (.45–2.85)	.790	3.91 (1.93–7.94)	** < .001**	.92 (.47–1.81)	.817	.69 (.26–1.85)	.457	1.96 (.85–4.52)	.116
**Lack of social support**	1.83 (1.09–3.07)	**.021**	2.10 (1.01–4.35)	**.046**	3.08 (1.33–7.17)	**.009**	1.00 (.55–1.82)	.995	1.26 (.54–2.92)	.596	1.20 (.43–3.35)	.724
**Feeling depressed**	1.63 (1.15–2.31)	**.006**	1.45 (.87–2.41)	.151	2.09 (1.12–3.92)	**.021**	.92 (.55–1.55)	.764	.87 (.40–1.89)	.726	.95 (.33–2.68)	.919
**Feeling anxious**	1.53 (1.07–2.20)	**.021**	1.28 (.77–2.11)	.336	2.01 (1.13–3.59)	**.017**	.94 (.54–1.64)	.820	1.01 (.47–2.19)	.981	1.39 (.58–3.32)	.463
**Concentration difficulties**	2.53 (1.83–3.51)	** < .001**	2.34 (1.48–3.67)	** < .001**	1.95 (1.06–3.60)	**.031**	1.96 (1.32–2.91)	** < .001**	2.35 (1.38–3.99)	**.002**	1.42 (.67–3.00)	.358
**Risk-taking behaviours and perceptions of risks**												
**Risk-taking behaviours**	1.74 (1.24–2.46)	**.002**	.41 (.24-.68)	** < .001**	.68 (.37–1.24)	.208	1.24 (.86–1.79)	.233	.40 (.24-.67)	** < .001**	.71 (.38–1.32)	.274
**Perceptions of risks**	1.44 (1.15–1.81)	**.002**	1.22 (.87–1.70)	.253	.79 (.50–1.24)	.299	1.25 (.98–1.59)	.068	1.19 (.82–1.71)	.357	.70 (.42–1.19)	.181

*Abbreviations: ADHD Attention-deficit/hyperactivity disorder, ASD Autism spectrum disorder, OR Odds ratio*

^*a*^*Unadjusted univariable model and*

^*b*^*Adjusted multivariable model for ADHD (n* = *182), ASD (n* = *78), the co-occurrence of both disorders (n* = *51), and 5.860 healthy controls*

## Discussion

This is the first-ever prospective study evaluating the impact of ADHD, ASD, and their co-occurrence on socio-economic, health, psychosocial vulnerabilities, risk-taking behaviours, and perceptions of risks in early adulthood. The observed associations suggested that socio-economic status, health quality, faith in the future, control over life, and social support are significantly compromised in individuals with any of these conditions. Our aim was especially to study the impact of the co-occurrence of both disorders, since it was shown that between 30 and 50% of individuals with ASD manifest ADHD symptoms, and two-thirds of individuals with ADHD show features of ASD [20].

The co-occurrence condition shared several morbidities with the ADHD group, thus both often experienced depressed and anxious feelings and worse sleep quality. Previous studies have found that as many as 80% of adults with ADHD have at least one coexisting psychiatric disorder [21, 22], including depression and anxiety, bipolar disorder, and substance use disorder (SUD) [23, 24]. Despite some studies also reporting higher lifetime rates of psychiatric comorbidities (major depressive disorder, anxiety, social phobia, and obsessive–compulsive disorder) in persons with autism [25–27], in this study the ASD group did not report depression or anxious feelings, neither higher perceived stress. Considering the association observed in this study between the co-occurrence and ADHD and serious life events, we could hypothesize that these individuals experienced, alongside the disorder-specific challenges, a more hostile environment (violence-related in the ADHD and more psychosocial in the co-occurrence), which can make them more prone to develop comorbid psychiatric symptomatology. In this line, it was also observed in this study that the co-occurrence group reported being bullied more often. Previous studies found that ADHD adults were more likely to have been divorced and less satisfied with their personal, social, and professional lives [28], while adults with autism often have satisfying social relationships [29]. Despite that, a lack of social support was reported as a common experience among all three conditions and could be attributed to social communication difficulties [30]. Interestingly the co-occurrence, but not each condition separately, was associated with a high risk of having an autoimmune disorder, which may suggest that both disorders could share the same autoimmune etiological mechanism. In previous studies celiac disease, ulcerative colitis, psoriasis, and T1D were linked to ADHD [31, 32], similarly, a study on adults on the spectrum reported a high prevalence of immune conditions (70.2%) in their sample [33].

In this study, individuals with ASD or those with the co-occurrence condition, were more likely to be unemployed, on this line, studies consistently reported unemployment rates around 30–40% in adults with autism [34, 35]. In accordance also with ASD, unlike the ADHD group, the co-occurrence was associated with lesser risk-taking behaviours and perceptions of risks. ADHD is accompanied by less activation of the frontoparietal networks associated with deficient inhibition, and impairments in executive functioning and decision-making [36], which may explain why this group perceived tobacco, e-cigarette, hashish/marijuana smoking, snuff use, and alcohol consumption as less harmful and therefore was more prone to these risk-taking behaviours. This same mechanism could also explain the association of ADHD with externalizing disorders such as conduct disorder and oppositional defiant disorder [37]. However, it could also be that drug use, through its pharmacological effects, make these persons less concerned with the consequences of their actions or more willing to become involved in risky behaviours or bad lifestyle to support a drug dependency or addiction [38]. Intriguingly, although there is a reported association between ADHD and overweight, we did not find a significant association, which may depend on the pharmacological treatment for ADHD, which is known to reduce appetite [39]. The co-occurrence however, showed a high risk of being underweight or overweight (similar to the ASD group). In the same direction as our results, some studies have reported higher rates of common chronic health conditions related to obesity, such as hypertension, dyslipidemia, diabetes, and in general poorer health outcomes in adults with autism [40]. Another study found self-rating general health as worse in a higher proportion of adults with autism [41]. In this study, despite all case-groups showing low health quality, the co-occurrence and ASD groups reported severe illness in the last two years in greater proportion than the control group did.

According to the results observed in this study, the individuals with ADHD seem to be exposed to different challenges than those with ASD. The ADHD group was characterized by more frequent somatic complaints (especially stomach and joint pain). In this line, a study found that adults with ADHD visited physicians 10 times more often and had rates of emergency room visits and hospitalization three times greater than controls [42]. This group also had worse job or academic feelings/performance, and lower physical activity. The ASD group also showed lower physical activity and longer screen exposure during weekends (> 4h/day). Establishing social relationships often comes with unique challenges for young-adults with ASD. One study found that subjects with autism who use social networking sites were found more likely to have close friends [43], which could explain the longer screen exposure of this group during weekends. A better understanding of the relative impact of these conditions in several areas of life could provide clues for enhanced specific-treatment options.

### Strengths and limitations

Our study has important strengths as our results are based on a large prospective birth cohort from the general population with a follow-up for more than 20 years and the strength of merging doctor-set diagnoses of ADHD, ASD, and autoimmune disorders via the National Diagnosis Register. However, our study also has some limitations. Besides diagnosis and household income, all other data are based on self-reported questionnaires, and therefore they could potentially be subject to recall bias, even though it is unlikely that this can explain our results. The attrition analyses showed that the families of young adults that responded to the 17–19 year questionnaire, have higher household income, higher parental education level, both parents were born in Sweden, and live together. If anything, this makes our observed associations even more obvious, suggesting that socio-economic status, health quality, faith in the future, control over life and social support are significantly compromised in individuals with any of these conditions. In addition, more young females than males participated, but we saw the same trends in both sexes. Future studies should gather information from sources beyond self-reports of individuals with ADHD and ASD, especially if they have psychiatric comorbidities. It might also be warranted to consider pharmacological treatment in subjects with ADHD in relation to different comorbidities.

## Conclusions

ADHD, ASD, and their co-occurrence significantly deteriorated socio-economic status, health quality, faith in the future, control over life, and social support. The co-occurrence of both disorders is a heavy burden for health, it is associated with several psychosocial vulnerabilities, and shares a similar morbidity pattern with ADHD while a less risk-taking behaviours and perceptions, according to the ASD group. Subjects with ADHD are exposed to different challenges than those with ASD. Understanding the impact of ADHD, ASD, and their co-occurrence allows improving the chance of prevention and development of early treatments with the potential to change the specific trajectory of morbidity later in life.

### Supplementary Information

**Additional file 1.**

## Data Availability

Deidentified participant data can be shared on reasonable request and ethical approval for a specified purpose, after approval by Johnny Ludvigsson (johnny.ludvigsson@regionostergotland.se) through a signed data access agreement.
