# Supplementary material for: Clinical implications of ADHD, ASD, and their co-occurrence in early adulthood—the prospective ABIS-study
Source: BMC Psychiatry. 2023 Nov 16;23:851. doi: 10.1186/s12888-023-05298-3 (PMC10655481; doi:10.1186/s12888-023-05298-3)
Supplement: Supplementary file 1 — Additional file 1. [file 12888_2023_5298_MOESM1_ESM.docx]

| **Appendix: Web survey with answer alternatives, 17-19 years of age follow up** | | | |
| --- | --- | --- | --- |
| **SOCIO-ECONOMIC INDICATORS** | **HEALTH-RELATED FACTORS** | **PSYCHOSOCIAL VULNERABILITY** | **RISK-TAKING BEHAVIORS AND PERCEPTIONS OF RISK** |
| Do you go to school? *[yes, no]*  Do you work professionally? *[yes, no]*  If not, I am? *[unemployed, other]*  Do you have enough money to generally do the same things as your friends? *[never, seldom, often, always]* | How good would you say your health is? *[excellent, very good, good, quite bad, bad]*  Have you experienced any of the following serious life events in the last two years? (Have been/are severely ill) *[yes, no]*  How much do you weight?  How tall are you?  Have you ever had a problem with allergies? *[yes, no]*  How often do you have headache? *[never, a couple of times per year, sometime per month, every week, almost every day]*  How often do you have stomach-ache? *[ never, a couple of times per year, sometime per month, every week, almost every day]*  How often do you have joint pain? *[never, a couple of times per year, sometime per month, every week, almost every day]*  Do you sometimes exercise so that you get sweaty? *[yes, no]*  How do you experience your sleep?  *[excellent, very good, good, quite bad, bad]*  How many hours a day on weekdays do you estimate sitting in front of a mobile screen/tablet? *[Never, 30m, 1- to 24h]*  How many hours a day on weekdays do you estimate sitting in front of a computer? *[Never, 30m, 1 to 24h]*  How many hours a day on weekends do you estimate sitting in front of a screen (computer, mobile screen, TV, tablet/iPad)? *[Never, 30m, 1 to 24h]* | Have you experienced any of the following serious life events in the last two years?   - Parent or sibling died *[yes, no]* - Mother or grandparent died *[yes, no]* - Severe illness in the family *[yes, no]* - Many conflicts between adults in the home *[yes, no]* - Divorce/parents who separated *[yes, no]* - Shared custody/alternating residence *[yes, no]* - Single custody (regular contact with the other parent) *[yes, no]* - Single custody (no or only sporadic contact with the other parent) *[yes, no]* - New adults in the family *[yes, no]* - New children in the family (also "bonus siblings") *[yes, no]* - Contact with support family *[yes, no]* - Contact social authorities for support *[yes, no]* - Foster home placement *[yes, no]* - Sexually abused (by an adult or peer) *[yes, no]* - Beaten/assaulted (by an adult or peer) *[yes, no]* - Robbery victim *[yes, no]*   How do you feel at school/work? *[from 1=Dislikes a lot to 10=thrives very well]*  How do you see your future? *[from 1=hopeless to 10=hopeful])*  How stressed have you felt the last month? *[from 1=not stressed at all to 10=very stressed]*  How well do you feel you have control over your life? *[from 1=no control to 10=full control]*  Do you feel bullied? *[never, seldom, sometimes, often, always]*  Do you feel you have the support from the environment (family, friends, school) that you need? *[yes, no]*  How often do you feel depressed? *[never, a couple of times per year, sometime per month, every week, almost every day]*  How often do you feel worried/anxious? *[never, a couple of times per year, sometime per month, every week, almost every day]*  How often do you experience concentration difficulties? *[never, a couple of times per year, sometime per month, every week, almost every day]* | Do you smoke? *[yes, no]*  Have you ever smoked e-cigarettes? *[yes, no]*  Have you ever smoked hashish/marijuana? *[yes, no]*  Do you use snuff? *[yes, no]*  Do you drink alcoholic beverages? *[yes, no]*  Imagine that someone smokes 2-3 times per day, how harmful do you think it is for health? *[not harmful at all, slightly harmful, moderately harmful, quite harmful, extremely harmful]*  Imagine that someone smokes e-cigarettes with nicotine 2-3 times per day, how harmful do you think it is for health? *[not harmful at all, slightly harmful, moderately harmful, quite harmful, extremely harmful]*  Imagine that someone smokes hashish/marijuana 2-3 times per week, how harmful do you think it is for health? *[not harmful at all, slightly harmful, moderately harmful, quite harmful, extremely harmful]*  Imagine that someone sniffs 2-3 times per day (one dose per week), how harmful do you think it is for health? *[not harmful at all, slightly harmful, moderately harmful, quite harmful, extremely harmful]*  Imagine that someone drinks a bottle of wine (three cans of 50 cl strong beer) on one occasion, every week, how harmful do you think it is for health? *[not harmful at all, slightly harmful, moderately harmful, quite harmful, extremely harmful]* |
